# Supplementary material for: Inhibition of NADPH Oxidases Prevents the Development of Osteoarthritis
Source: Antioxidants (Basel). 2022 Nov 27;11(12):2346. doi: 10.3390/antiox11122346 (PMC9774355; doi:10.3390/antiox11122346)

Supplementary Table S1. Primer list

Gene	Primer	Primer sequences
NOX1	forward	AGG TCG TGA TTA CCA AGG TTG TC
	reverse	AAG CCT CGC TTC CTC ATC TG
NOX2	forward	AGC TAT GAG GTG GTG ATG TTA GTG G
	reverse	CAC AAT ATT TGT ACC AGA CAG ACT TGA G
NOX3	forward	ACC GTG GAG GAG GCA ATT AGA CAA
	reverse	TTC CAG GTT GAA GAA ATG CGC CAC
NOX4	forward	CCC AAG TTC CAA GCT CAT TTC C
	reverse	TGG TGA CAG GTT TGT TGC TCC T
Vegf	forward	GAG AGA GGC CGA AGT CCT TT
	reverse	TTG GAA CCG GCA TCT TTA TC
Fth1	forward	TGA CCA CGT GAC CAA CTT ACG
	reverse	CCA GGG TGT GCT TGT CAA AGA
Hmox	forward	TGA CCA CGT GAC CAA CTT ACG
	reverse	CCAGGGTGTGCTTGTCAAAGA
Txn	forward	ATC AAG CCC TTC TTC CAT TCC
	reverse	TCC TGG CAG TCA TCC ACA TC
Arg	forward	ACA TCA ACA CTC CCC TGA CAA
	reverse	TAC GTC TCG CAA GCC AAT GTA
Adamts5	forward	CCA TCT TCC CGG TTG TGT ATC T
	reverse	ACT GTT TCA CTC TGG GCT CTT
MMP13	forward	GGA CTC ACT GTT GGT CCC TG
	reverse	GGA TTC CCG CAA GAG TCA CA
Gapdh	forward	AGC CCA AGA TGC CCT TCA GT
	reverse	CCG TGT TCC TAC CCC CAA TG

Supplementary Table S2. Antibody list

Antibody	Host	Dilution	Application	Source	Catalogue number
Adamts5	Rabbit	1:1000	IB	Abcam, Cambridge, UK	ab41037
Akt	Rabbit	1:1000	IB	Cell Signaling Technology, Beverly, MA, USA	#9272
p-Akt	Rabbit	1:1000	IB	Cell Signaling Technology, Beverly, MA, USA	#9271
Aggrecan	Rabbit	1:1000	IB	Abcam, Cambridge, UK	ab36861
β-actin	Mouse	1:5000	IB	Sigma-Aldrich, Burlington, MA, USA	A1978
IκBα	Mouse	1:1000	IB	Cell Signaling Technology, Beverly, MA, USA	#4814
p-IκBα	Mouse	1:1000	IB	Cell Signaling Technology, Beverly, MA, USA	#9246

MMP13	Rabbit	1:1000	IB	Abcam, Cambridge, UK	ab51072
NF-κB p65	Rabbit	1:1000	IB	Cell Signaling Technology, Beverly, MA, USA	#8242
p-NF-κB p65	Rabbit	1:1000	IB	Cell Signaling Technology, Beverly, MA, USA	#3033
p38 MAPK	Rabbit	1:1000	IB	Cell Signaling Technology, Beverly, MA, USA	#9212
p-p38	Rabbit	1:1000	IB	Cell Signaling Technology, Beverly, MA, USA	#4631
Stat3	Rabbit	1:1000	IB	Cell Signaling Technology, Beverly, MA, USA	#12640
p-Stat3	Rabbit	1:2000	IB	Cell Signaling Technology, Beverly, MA, USA	#9145
ERK	Mouse	1:5000	IB	BD Biosciences, Franklin Lakes, New Jersey , USA	610123
p-Erk	Rabbit	1:1000	IB	Cell Signaling Technology, Beverly, MA, USA	#9101
Collagen Ⅱ	Rabbit	1:1000	IB	Abcam, Cambridge, UK	ab34712
JNK	Rabbit	1:1000	IB	Cell Signaling Technology, Beverly, MA, USA	#9252
p-JNK	Rabbit	1:1000	IB	Cell Signaling Technology, Beverly, MA, USA	#9251
8-oxo-dG	Mouse	1:200	IHC	Santa Cruz Biotechnology, Santa Cruz, CA, USA	sc-66036
NOX1	Rabbit	1:1000,1:200	IB, IHC	Novusbiologicals, Novus, Briarwood Avenue, USA	NBP1-31546
NOX2	Mouse	1:1000,1:200	IB, IHC	Invitrogen, Waltham, Massachusetts, USA	MA5-18052
NOX3	Rabbit	1:1000,1:200	IB, IHC	Invitrogen, Waltham, Massachusetts, USA	# PA5-38036
NOX4	Rabbit	1:1000,1:200	IB, IHC	Novusbiologicals, Novus, Briarwood Avenue, USA	NB110-58849
FITC-conjugated anti-rabbit	Donkey	1:200	IHC	Jackson Immuno Research Laboratories	711-096-152
Cy3-conjugated anti-mouse	Donkey	1:200	IHC	Jackson Immuno Research Laboratories	715-165-151
HPR-linked anti-rabbit	Donkey	1:2000	IB	Cell Signaling Technology, Beverly, MA, USA	7074s
HPR-linked anti-mouse	Donkey	1:2000	IB	Cell Signaling Technology, Beverly, MA, USA	7076s

Biotinylated anti-mouse	Goat	1:200	IHC	Vector, Laboratories, Burlingame, CA, USA	BA-2000
Biotinylated anti-rabbit	Goat	1:200	IHC	Vector, Laboratories, Burlingame, CA, USA	BA-1000

IHC: immunohistochemistry; IB: immunoblot

Supplementary Table S3. The source data of Figure 2D. The positive cells for NOX isoform and DAPI positive cells were counted with Image J program, and the proportion of NOX positive cells were calculated.

Number of NOX positive cells

	NOX1					NOX2					NOX3					NOX4				
0	68	62	76	78	65	43	31	35	33	45	80	77	72	83	73	3	6	14	12	10
2	44	37	31	38	40	74	72	66	63	69	13	24	33	21	19	33	46	48	32	39
4	24	24	37	42	30	84	86	77	84	87	21	34	12	38	24	53	59	58	47	54
8	33	48	31	34	36	80	87	72	83	73	35	23	26	38	26	73	74	76	75	68

(Weeks)

Number of DAPI positive cells

	NOX1					NOX2					NOX3					NOX4				
0	213	245	215	211	200	214	227	214	224	213	219	231	229	213	202	221	208	245	215	215
2	211	235	206	209	224	250	205	203	210	208	240	221	209	232	236	227	250	249	240	222
4	214	238	232	222	227	217	237	244	245	205	216	200	250	248	245	201	232	206	244	201
8	217	212	208	211	228	217	205	204	232	218	249	237	247	231	210	214	235	246	224	241

(Weeks)

Number of positive cells (% of DAPI+)

	NOX1					NOX2					NOX3					NOX4				
0	68	62	76	78	65	43	31	35	33	45	80	77	72	83	73	3	6	14	12	10
2	44	37	31	38	40	74	72	66	63	69	13	24	33	21	19	33	46	48	32	39
4	24	24	37	42	30	84	86	77	84	87	21	34	12	38	24	53	59	58	47	54
8	33	48	31	34	36	80	87	72	83	73	35	23	26	38	26	73	74	76	75	68

(Weeks)

Supplementary Figure S1. Source data of Figure 3A and 3B

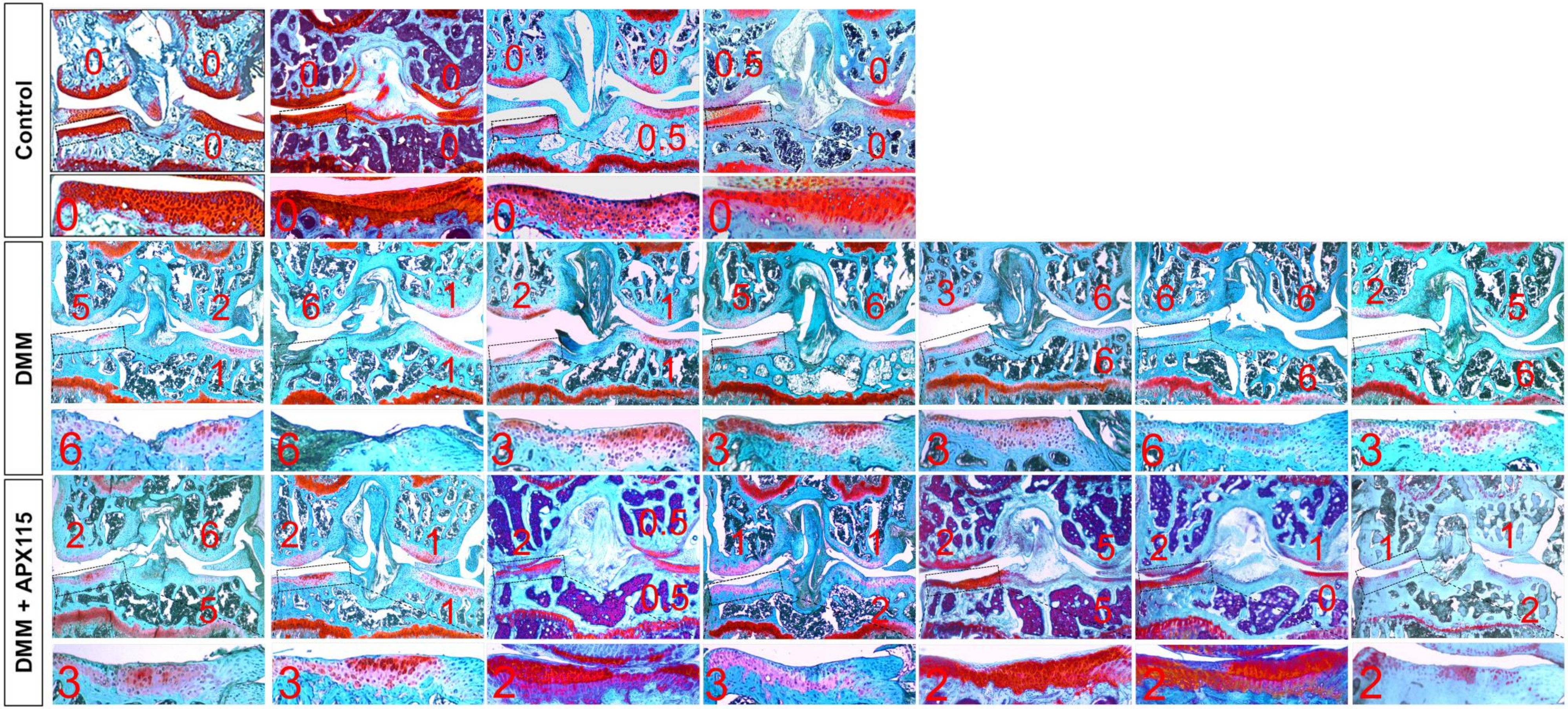

Supplement: Supplementary file 1 [file antioxidants-11-02346-s001.zip › antioxidants-2057165-supplementary.pdf]
